# 

**Published:** 1895-05

**Authors:** 


### CONTENTS.

ORIGINAL ARTICLES:
Horseshoeing. By R. S. HUIDEKOPER, M.D. . . . . . . . . . 269
The Anatomy of the Large American Fluke (*Fasciola Magna*) and a Comparison with Other Species of the Genus Fasciola, S.ST. By CHAS. WARDELL STILES, PH.D. . . . . . . . . . . . . . . 277
Antiseptic Surgery. By H. A. SPENCER, SR., V.S. . . . . . . . 283
Physiology of the Kidney. By H. BUSMAN, Student . . . . . . . 288
Distemper in Dogs. By W. H. MARTENET, D.V.S. . . . . . . . 292
EDUCATIONAL:
Synopsis of Address of Dean McEachran to the McGill Graduating Class . . 298
Synopsis of President's Address of California State Veterinary Medical Association 301
REPORTS OF CASES:
Bilateral Pneumonia—Flatulent Colic. By OTTO NOACK . . . . . . 302
Immobility following Influenza. By W. HORACE HOSKINS . . . . . . 304
LEGISLATION: New York—Pennsylvania—Massachusetts—Missouri—Kansas . 306
COMMENCEMENT EXERCISES:
Chicago Veterinary College—McGill University—National Veterinary College—U. S. College of Veterinary Surgeons . . . . . . . . . . 310
EDITORIALS:
Opposition to State Examiners in Ohio—Associated Veterinary Faculties—Contagious Pleuro-pneumonia—Veterinary Columns in Secular Journals—The National Cat-Show—The United States College and the Three-years' Course 315
OBITUARY—Joseph Gamgee . . . . . . . . . . . . . 319
REVIEW—A Book on Cats . . . . . . . . . . . . . . 320
SELECTIONS:—American Horseflesh in European Food-markets—Separator Skim-milk—Dr. Louis Waldstein and the Pilocarpine Treatment of Tuberculosis and Cancer—No Contagious Pleuro-pneumonia in Kansas . . . . . . . 321
SOCIETY PROCEEDINGS:
The International Veterinary Congress at Berne, Switzerland—Veterinary Medical Association of New York County—Keystone Veterinary Medical Association—District of Columbia Veterinary Medical Association—Illinois Veterinary Medical Association—Massachusetts State Veterinary Medical Association—Veterinary Medical Association, State of New Jersey . . . . . . 328
PERSONALS—U. S. V. M. A.—CORRESPONDENCE . . . . . . . 332–336

